# Infection- and procedure-dependent effects on pulmonary gene expression in the early phase of influenza A virus infection in mice

**DOI:** 10.1186/1471-2180-13-293

**Published:** 2013-12-17

**Authors:** Matthias Preusse, Mohamed A Tantawy, Frank Klawonn, Klaus Schughart, Frank Pessler

**Affiliations:** 1 Institute for Experimental Infection Research, TWINCORE Center for Experimental and Clinical Infection Research, Feodor-Lynen-Str. 7, Hannover 30625, Germany; 2 Department of Cellular Proteomics, Bioinformatics, Helmholtz Centre for Infection Research, Inhoffenstrasse 7, Braunschweig 38124, Germany; 3 Department of Infection Genetics, Helmholtz Centre for Infection Research, Inhoffenstrasse 7, Braunschweig 38124, Germany; 4 Helmholtz Centre for Infection Research, Inhoffenstrasse 7, Braunschweig 38124, Germany

**Keywords:** Anesthesia, Gene expression, Influenza virus, Interferon, Interferon lambda, Intranasal infection, Mouse model

## Abstract

**Background:**

Investigating the host response in the early stage of influenza A virus (IAV) infection is of considerable interest. However, it is conceivable that effects due to the anesthesia and/or intranasal infection procedure might introduce artifacts. We therefore aimed to evaluate the effects of anesthesia and/or intranasal infection on transcription of selected pulmonary mRNAs in two inbred mouse strains with differential susceptibility to IAV infection.

**Results:**

DBA/2J and C57BL/6J mice were evaluated in a time course experiment in which lung tissue was sampled after 6, 12, 18, 24, 48 and 120 h. After anesthesia with ketamine and xylazine, a suspension of mouse-adapted IAV strain PR8_Mun in 20 μl sterile buffer, or 20 μl sterile buffer only, was instilled intranasally. The mice receiving anesthesia and PBS only were designated the “mock treatment” group. Pulmonary expression of 10 host mRNAs (*Fos*, *Retnla*, *Irg1*, *Il6*, *Il1b*, *Cxcl10*, *Stat1*, *Ifng*, *Ifnl2*, and *Mx1*) and viral hemagglutinin (HA) mRNA were determined at the designated time points. As expected, weight loss and viral replication were greater in the DBA/2J strain (which is more susceptible to IAV infection). Four mRNAs (*Retnla*, *Irg1*, *Il6*, and *Cxcl10*) were procedure-dependently regulated in DBA/2J mice between 6 and 24 h, and two (*Retnla* and *Il6*) in C57BL/6J mice, although to a lesser extent. All 10 mRNAs rose after infection, but one (*Fos*) only in DBA/2J mice. These infection-dependent effects could be separated from procedure-dependent effects beginning around 12 h in DBA/2J and 18 h in C57BL/6J mice. The interferon-related mRNAs *Stat1, Ifng*, *Infl2,* and *Mx1* were unaffected by mock treatment in either mouse strain. *Mx1* and *Infl2* correlated best with HA mRNA expression (r = 0.97 and 0.93, respectively, in DBA/2J).

**Conclusions:**

These results demonstrate effects of the anesthesia and/or intranasal infection procedure on pulmonary gene expression, which are detectable between approximately 6 and 24 h post procedure and vary in intensity and temporal evolution depending on the mouse strain used. Mock infection controls should be included in all studies on pulmonary gene expression in the early phase of infection with IAV and, likely, other respiratory pathogens.

## Background

Due to ease of infection, animal rearing, and the availability of genetically modified strains, using mouse models and viral strains adapted to the murine host has become an attractive approach to studying the mammalian response to influenza A virus (IAV) infection. Recently, a substantial amount of information has been obtained regarding gene expression changes at various stages of infection in this model [1-3]. These authors showed that the genetic background of different mouse strains strongly influences the susceptibility to IAV. For instance, DBA/2J mice were highly susceptibility to the PR8_Mun IAV (H1N1) strain, whereas C57BL/6J mice were more resistant, as evidenced by less weight loss and an approx. 1000-fold higher viral LD_50_. Conversely, viral load was significantly higher in the DBA/2J strain, which also mounted a hyper-inflammatory response with much stronger up-regulation of many immune response-dependent genes. As exemplified by the aforementioned studies, most work in murine models of IAV infection has focused on time points during or after established infection (1 day up to 60 days), and very little attention has been paid to the first 24 hours (h). Nevertheless, critical aspects of the host response to early steps in viral attachment and entry could conceivably be studied during this early time window. However, due to the temporal proximity to the technical and pharmacological manipulations surrounding the infection process, it is conceivable that both the administration of the anesthetic and the physical and physiological stress from intranasal installation of the inoculate would lead to artifactual signals that are unrelated to the virus-host interaction. We have therefore analyzed changes in pulmonary gene expression in a 5-day time course featuring frequent measurements in the first 24 h, comparing results obtained from mice infected with IAV or exposed to vehicle only (“mock infection”). We find effects on pulmonary gene expression that can be clearly ascribed to the anesthesia/infection procedure, which are detectable as early as 6 h post treatment and differ between the two mouse strains in terms of magnitude and temporal evolution.

## Methods

### Sample preparation

Female 12-13-week-old C57BL/6J and DBA/2J mice (n = 5–8 per time point and treatment) and mouse-adapted IAV strain variant PR8_Mun (Institute of Molecular Virology, University of Muenster, Germany), which is closely related to A/Puerto Rico/8/34, were used. Mice were weighed on day 0 just before induction of anesthesia and on each subsequent day. Infections were essentially carried out as described previously [1]. Briefly, mice were anesthetized by intra-peritoneal injection of 10 μl per g body weight of a stock solution of 0.5 ml ketamine (50 mg/ml, Invesa Arzneimittel GmbH, Freiburg, Germany), 0.5 ml 2% xylazine hydrochloride (Bayer Health-Care, Leverkusen, Germany) and 9 ml sterile NaCl 0.9% (Delta-Select GmbH, Dreieich, Germany). For intranasal infection, a viral dose of 2 × 10^3^ focus forming units (ffu) of PR8_Mun (propagated in embryonated chicken eggs) was administered in a total volume of 20 μl sterile phosphate-buffered saline (PBS). During the infection procedure, mice were held in the upright position and additional anesthetic was reinjected as needed. Mock treatment was identical to real anesthesia/infections except that vehicle only (sterile PBS), not containing virus, was used for intranasal instillation. Mice were killed by CO_2_ asphyxiation at 6, 12, 18, 24, 48, and 120 h with respect to infection or mock treatment. Untreated mice were used as t = 0 h control. Lungs were removed from the cadavers by cutting the main bronchus and were washed in RNAlater RNA Stabilization Reagent (Qiagen Inc, Valencia, CA, USA) immediately after removal. After transfer into a new tube containing 2 ml RNAlater, lungs were stored overnight at 4°C and then at -20°C until further use. All animal work was approved by an external committee according to the regulations on animal welfare of the Federal Republic of Germany.

### RNA isolation and qRT-PCR

Lungs were homogenized in 4 ml RLT buffer (Qiagen) containing 40 μl β-mercaptoethanol and stored at -80°C in 450 μl aliquots. After thawing, 450 μl of this suspension was mixed with 700 μl Qiazol (Qiagen), and all further steps of total RNA isolation were performed with the miRNeasy kit (Qiagen) according to the manufacturer’s recommendations. Real-time RT-PCR (qRT-PCR) was performed with a LightCycler 480 (La Roche AG, Basel, Switzerland) in 96 well plates in 20 μl reaction volumes, using 15 ng cDNA (miScript Reverse Transcription Kit, QuantiTect SYBR Green PCR Kit) and primers specific for the following targets: the immediate early gene FBJ osteoscarcoma oncogene (*Fos)*, resistin like α (*Retnla)*, immune-responsive gene 1 (*Irg1)*, interleukin 6 (*Il6)*, interleukin 1β (*Il1b)*, the chemokine (C-X-C motif) ligand 10 (*Cxcl10)*, four genes related to interferon pathways (the transcription factor signal transducer and activator of transcription 1 (*Stat1)*, interferon γ (*Ifng)*, interferon λ2 (*Ifnl2,* aka *Il28a)*, and myxovirus (influenza virus) resistance 1 (*Mx1)),* and IAV hemagglutinin (HA*)*. Quantitect Primer Assays (Qiagen) were used for all targets except *Ifnl2* and HA. Primers for amplification of *Ifnl2* were designed using exon-spanning regions of the NCBI [4] sequence (Tanta_Mus_Ifnl2-F: 5’ctgcttgagaaggacctgagg’3, Tanta_Mus_Ifnl2-R: 5’ctcagtgtatgaagaggctggc’3). Primer sequences for HA mRNA amplification were published previously [3]. Mouse Genome Informatics (MGI) gene symbols and names were used for all genes [5]. The arithmetic mean of the Ct values of β actin (*Actb*) and ribosomal protein L4 (*Rpl4)* was used as internal reference for normalization.

### Data analysis

Data were analyzed using the R environment and programming code [6]. qRT-PCR data points with C_t_ ≥40, corresponding to lack of detection of a target due to technical failure or lack of expression, were assigned a C_t_ of 40. We removed technical outliers in ΔC_t_ values using the maximum normed residual test (Grubbs’ test) to detect outliers for each condition with a threshold of p ≤0.05. A median of 5 (range, 3–8) biological replicates were available for each data point after outlier removal. ANOVA was used for testing of trends throughout time series, adjusting p values for false discovery rate (FDR). For pairwise comparisons, we used Tukey’s Honest Significant Differences Test for homogeneous variances and Dunnett’s Modified Tukey-Kramer Pairwise Multiple Comparison Test for heterogeneous variances (Levene’s test for variance equality). We used a significance threshold of p ≤0.05.

## Results

### Changes in body weight

**Figure 1:**
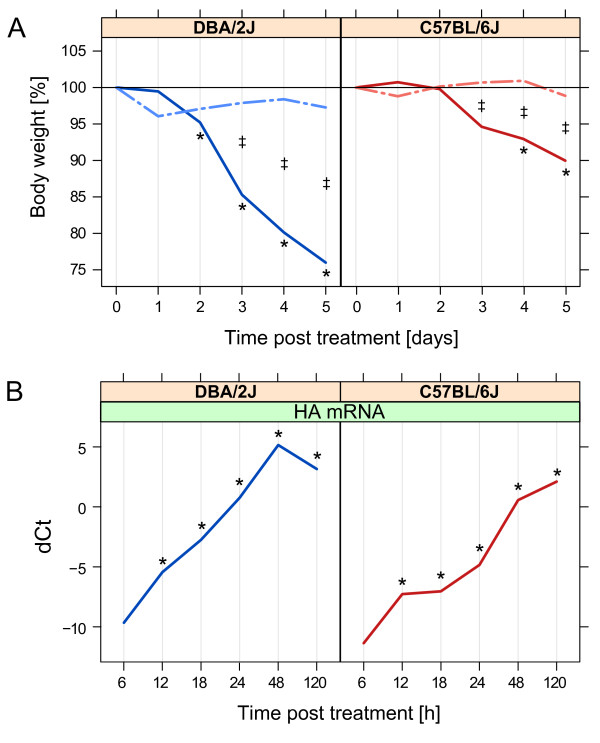
**Weight loss and expression of IAV HA mRNA throughout the 5-day time course after mock treatment or infection with IAV strain PR8_MUN as outlined in the Methods section. A**. Weight loss, expressed as the percentage of body weight measured at t = 0 h before administration of anesthesia. No mice had to be killed because of >30% weight loss. **B**. Relative quantification of IAV HA mRNA in mouse lung by qRT-PCR in the 5-day time course shown in panel A. dCt refers to Ct_reference_ – Ct_target mRNA_, where Ct_reference_ corresponds to the arithmetic mean of the Ct values of *Actb* and *Rpl4*. Solid lines, infection; interrupted lines, mock treatment. Left panels, DBA/2J strain; right panels, C57BL/6J strain. Note that the x-axes of the panels are based on different scales. *, p ≤0.05 for difference with respect to t = 0 h; ‡, p ≤0.05 for difference between mock-treated and infected mice at the given time point (Tukey’s test).

Weight loss in infected DBA/2J mice became manifest by day 2 (Figure 1A) and reached approx. 25% by day 5. As shown previously [3], weight loss in the infected C57BL/6J mice was less pronounced, as reflected in a mere 5 - 10% reduction by day 4 – 5. In both strains, statistically significant differences between infected and mock treated mice were observed by day 3. Mock-treated mice showed no significant weight loss at any time point. Thus, there was no significant effect of the anesthesia/infection procedure on body weight in either mouse strain.

### Viral replication

qRT-PCR revealed a brisk rise of mRNA encoding IAV HA in lungs of both mouse strains after infection (Figure 1B). HA mRNA was detected at low levels as early as 6 h in both strains, followed by a rapid rise that peaked at 48 h and 120 h in DBA/2J and C57BL/6J mice, respectively. HA mRNA levels were significantly higher in DBA/2J than in C57BL/6J starting around 12 h. As expected, HA mRNA was not detected in the mock treated mice.

### Principle component analysis of pulmonary expression of host-encoded mRNAs

**Figure 2:**
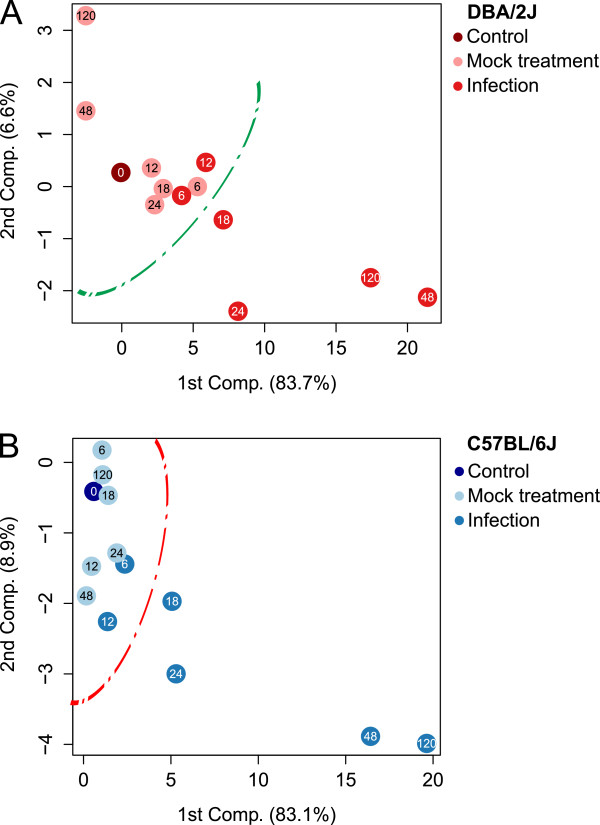
**Principal component analysis of pulmonary expression of the 10 host-encoded mRNAs in mock-treated and infected DBA/2J and C57BL/6J mice in the 5-day time course of IAV infection.** mRNA levels of *Fos*, *Retnla*, *Irg1*, *Il6*, *Il1b*, *Cxcl10*, *Stat1*, *Ifng*, *Ifnl2*, and *Mx1* were determined by qRT-PCR as outlined in the Methods section, using *Actb* and *Rpl4* mRNA expression for internal normalization. Each dot refers to the mean value of mice of one treatment as outlined in the legend adjacent to the box. The number inside each dot identifies the time (h) elapsed since t = 0 h. **A**. Results obtained with the DBA/2J strain. **B**. Results obtained with the C57BL/6J strain. Including the third component did not lead to further discrimination (data not shown).

A cluster containing infected and mock treated time points could be identified easily in both mouse strains (Figure 2). A separation between infected and mock-treated samples became evident at 18 h in both mouse strains, as indicated by the lines in Figure 2. Marked step-offs between 24 and 48 h were seen in both strains. Consistent with the continuing rise of HA mRNA in the C57BL/B6 strain between 48 and 120 h the 120 h time point localized beyond the 48 h time point. In contrast, in the DBA/2J strain HA mRNA declined between 48 and 120 h, and the 120 h time point localized between 24 and 48 h in the PCA plot. In both strains, the t = 48 h and 120 h mock treated mice localized far away from the infected t = 48 and 120 h mice. Thus, this analysis showed that in both mouse strains procedure-related effects did not contribute significantly to the gene expression changes observed in infection beyond 24 h and that the evolution of gene expression reprogramming correlated well with transcription of HA mRNA in both strains.

### Pulmonary expression of individual host-encoded mRNAs

**Figure 3:**
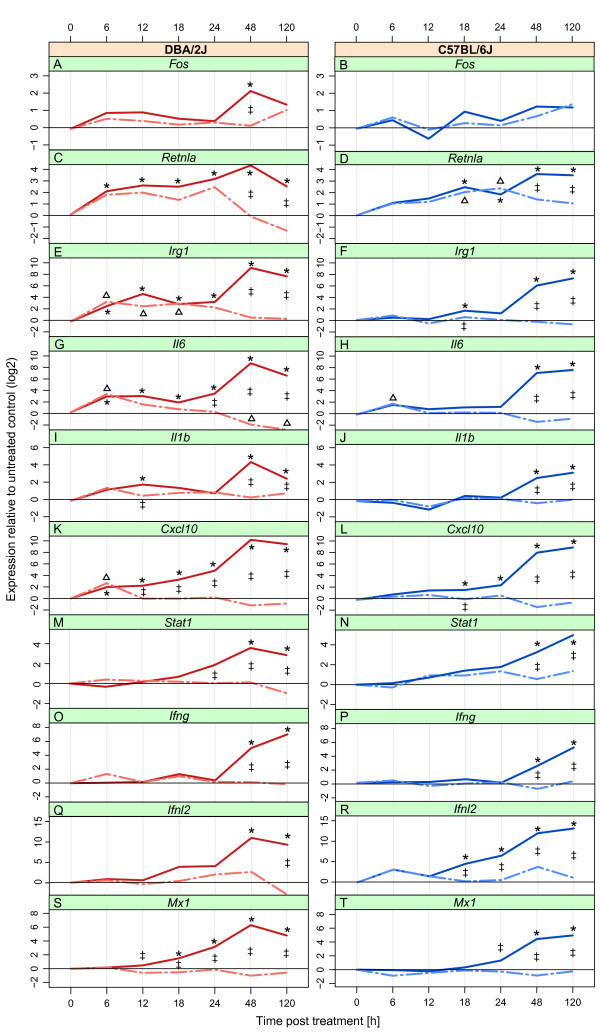
**Expression changes in mock-treated and infected DBA/2J (left column) and C57BL/6J (right column) mice in the 10 target host mRNAs in the 5-day time course of IAV infection.** Analysis of the same data set as used for Figure 2. Panels show fold change expression, determined by qRT-PCR, of *Fos***(A, B)**, *Retnla***(C, D)**, *Irg1***(E, F)**, *Il6***(G, H)**, *Il1b***(I, J)**, *Cxcl10***(K, L)**, *Stat1***(M, N)**, *Ifng***(O, P)**, *Ifnl2***(Q, R)**, and *Mx1***(S, T)** mRNA. Fold change data of *Ifnl2* represent an underestimation, as a Ct of 40 was assigned to all samples with Ct >40 (see Methods section). Solid lines, mice intranasally infected with 1x10^3^ ffu of IAV strain PR8_Mun; interrupted lines, mice undergoing the same anesthesia/infection procedure except that buffer only, not containing virus, was used for intranasal installation (mock treatment). *, p ≤0.05 for difference between infected mice at the given time point with respect to t = 0 h; ∆, p ≤0.05 for difference between mock treated and control (t = 0 h) mice; ‡, p ≤0.05 for difference between infected and mock treated mice at the given time point. All p values were determined with Tukey’s test.

Results are shown in Figure 3. All 10 host mRNAs exhibited at least some evidence of regulation throughout the time course (ANOVA). Four mRNAs were also significantly regulated in response to mock treatment, but two of these (*Cxcl10* and *Irg1*) were regulated only in the DBA/2J strain. *Fos*, *Il1b*, *Stat1, Ifng*, *Ifnl2*, and *Mx1* mRNAs were not regulated by mock treatment in either strain.

*Fos* mRNA was expressed at low level and increased significantly after 48 h in the infected DBA/2J mice only (panel A). There was no evidence for mock treatment-dependent regulation of this mRNA in either mouse strain (panels A and B). The apparent tendency of an increase in infected C57BL/6J mice toward the later time points was not significant. *Retnla* mRNA increased significantly at all time points in infected DBA/2J mice. ANOVA revealed a significant effect of the mock treatment, with an effect size resembling that of infection between 6 and 24 h, suggesting procedure-dependent regulation through 24 h and infection-dependent regulation thereafter (panel C). In infected C57BL/6J mice, *Retnla* increased between 18 h and 120 h (panel D). In mock treated mice of this strain, expression increased steadily at the early time points, with significant regulation at 18 h and 24 h. This suggested a procedure-dependent regulation of *Retnla* resembling that observed in the DBA/2J mice. *Irg1* mRNA expression increased in mock-treated DBA/2J mice between 6 and 18 h (panel E). This gene was up-regulated in DBA/2J infected mice at all time points, reaching a maximal 630-fold induction on day 2. In the C57BL/6J strain there was no increase in *Irg1* due to mock treatment, and the infection-dependent increase was less pronounced, reaching a max. 150-fold induction at 120 h. *Il6* mRNA increased in both strains beginning 6 h after infection or mock treatment, with stronger regulation being observed in the DBA/2J mice (panel G). In DBA/2J mice the mock treatment effect declined towards 18 h, and clear differences between infected and mock treated mice became apparent at 24 h. In the C57BL/6J mice, an infection-dependent rise in *Il6* mRNA was observed somewhat later (t = 48 h) (panel H). *Il1b* mRNA increased in infected mice of both strains at 48 h and 120 h, and there was a tendency (p at 6 h = 0.09) toward a mock treatment effect between 6 and 18 h in the DBA/2J strain (panel I). *Cxcl10* mRNA was up-regulated in DBA/2J mock-treated mice at 6 h (panel K), whereas it was not affected by mock treatment in the C57BL/6J mice (panel L). In both mouse strains *Cxcl10* mRNA was significantly elevated in the infected mice, beginning at 6 h in the DBA/2J and at 18 h in C57BL/6J. *Stat1* expression was not affected by mock treatment in DBA/2J mice, but there was a slight trend (statistically not significant) for up-regulation in C57BL/6J mice. An infection-dependent up-regulation became apparent at 24 h and 48 h in DBA/2J and C57BL/6J mice, respectively. Similar to *Stat1*, *Ifng* was up-regulated in both mouse strains beginning around 48 h, and there was no evidence for regulation due to the infection procedure. *Ifnl2* was not detected (C_t_ ≥ 40) in about 40% of untreated and mock treated DBA/2J mice; fold change values therefore represent an underestimation (panel Q). A significant rise after infection became apparent at 48 h, reaching a mean C_t_ of 26.3. In C57BL/6J mice, it was not detected in about 80% of the untreated and mock treated samples, suggesting a lower baseline expression than in DBA/2J (panel R). A first significant infection-dependent regulation was observed at 18 h, where *Ifnl2* was detected in all DBA/2J and four of five C57BL/6J samples. *Ifnl2* was detected in all 24 h samples (C_t_ = approx. 33) and continued to rise through 120 h. There was no evidence for a mock treatment effect on *Ifnl2* in either mouse strain. *Mx1* mRNA expression (panels S and T) was not regulated in response to mock treatment in either strain. In contrast, it strongly increased during infection, showing a significant rise beginning at 12 h in DBA/2J and 24 h in C57BL/6J mice.

Taken together, these results allow classifying the analyzed genes into three groups: (1) genes that were regulated in response to mock treatment and infection in both strains (*Retnla, Il6*), (2) genes that were regulated in response to both mock treatment and infection in the DBA/2J strain only (*Irg1*, *Cxcl10*), and (3) those whose expression changed in response to infection only (*Fos*, *Il1b*, *Stat1*, *Ifng*, *Ifnl2*, and *Mx1*). Of note, the latter group contained all four interferon pathway-related mRNAs.

### Correlation with IAV HA mRNA

Expression of the 10 host mRNAs was then correlated with HA mRNA expression (Table 1). Overall, correlations were higher in the DBA/2J strain. Only *Il1b* correlated more strongly in C57BL/6J than in DBA/2J. *Mx1* and *Ifnl2* mRNA levels correlated best with HA mRNA expression in both strains, whereas *Fos* mRNA was the only one that did not correlate with HA mRNA.

**Table 1:** **Correlations of pulmonary expression of 10 target mRNAs with HA mRNA**^**1**^

mRNA	DBA/2J	C57BL/6J
*Mx1*	0.97***	0.89***
*Ifnl2*	0.93***	0.87***
*Cxcl10*	0.92***	0.87***
*Stat1*	0.90***	0.86***
*Il6*	0.80***	0.68***
*Ifng*	0.70**	0.62**
*Irg1*	0.76***	0.72***
*Retnla*	0.62**	0.63***
*Il1b*	0.53*	0.71***
*Fos*	0.39	0.16

^1^Values correspond to Spearman correlation coefficient in mouse strains infected with IAV, sorted by decreasing values in DBA/2J mice. P values (FDR adjusted): ***, ≤0.001; **, ≤0.01; *, ≤0.05.

### Regulation across all 10 target mRNAs

**Figure 4:**
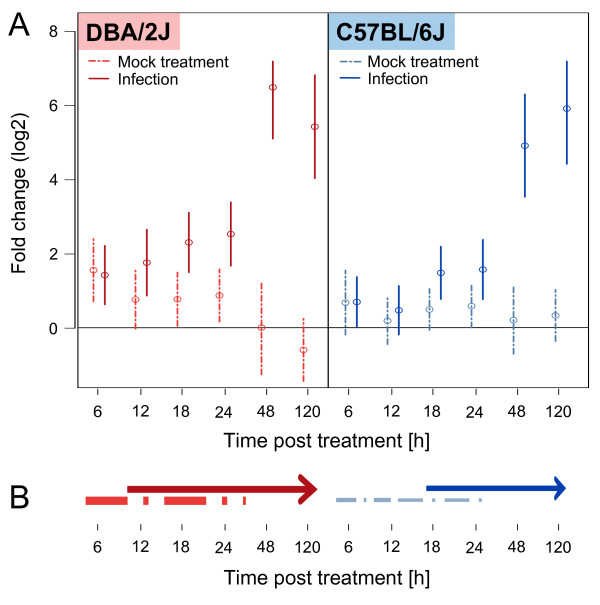
**Overall mean fold changes in mRNA expression throughout the time course. A**. Mean expression changes in mock-treated and infected DBA/2J and C57BL/6J mice across all 10 target host mRNAs in the 5-day time course of IAV infection. The analysis is based on the same data set as used for Figures 2 and 3. Mean fold change values and 95% confidence intervals (vertical lines) were calculated with the Dunnett’s Modified Tukey-Kramer test, using the dCt values (qRT-PCR) of all 10 host-encoded mRNAs as input. **B**. Schematic representation of the results shown in panel A. As reflected by the thickness of the lines, overall changes are more pronounced in the DBA/2J strain. Procedure-dependent effects are evident between 6 and 24 h in both strains, but infection-related changes begin to evolve and peak earlier in the DBA/2J strain.

Results are summarized in Figure 4. Considering regulation across all 10 target mRNAs combined, we detected a significant up-regulation at all time points after 0 h in infected DBA/2J mice (Dunnett’s Modified Tukey-Kramer Pairwise Multiple Comparison Test). Among mock treated DBA/2J mice, an up-regulation was observed at 6, 18 and 24 h post treatment. The strongest effect was detected at 6 h (mean fold increase, 2.9; CI = 1.6-5.4) which nearly equaled the regulation in infected mice (mean fold increase, 2.7; CI = 1.5-4.7). A significant difference between infected and mock-treated DBA/2J mice could be discerned by ANOVA beginning at 12 h, but a contribution of a procedure-related effect to mRNA expression in the infected mice could be excluded only from 48 h onward. Messenger RNA up-regulation peaked at 48 h and began to decline by 120 h. In the C57BL/6J strain, overall up-regulation was less than in the DBA/2J strain. In this strain, the expression change at 6 h seemed to be due to the anesthesia/infection procedure in both infected and mock-treated mice, as fold induction was nearly identical in both (mean fold induction, 1.6; CI_Inf_ = 0.98-2.6 and CI_Mock_ = 0.84-2.9). As in the DBA/2J strain, a procedure-dependent effect seemed to persist through 24 h (CI_Mock_ = 0.97-2.23). Infection-dependent mRNA up-regulation first became manifest at 18 h and continued to rise between 48 and 120 h. Thus, this analysis supported the kinetics of evolution of infection-dependent transcription in both mouse strains, as revealed by the PCA (Figure 2). In addition, consistent with the results shown in Figure 3, it showed that procedure-dependent effects occurred before 48 h and were more pronounced in the DBA/2J strain.

## Discussion

This analysis of sequential changes in pulmonary expression of several mRNAs after real or simulated IAV infection revealed effects that can be ascribed to anesthesia and/or the intranasal inoculation procedure. The results clearly demonstrate that the appropriate control group treated with a simulated anesthesia/infection should always be included in studies of IAV infection in mice that cover approximately the first 24 h post infection.

What might be the underlying pathophysiological mechanisms? Anesthesia is known to influence cytokine expression in humans, but actually appears to have an anti-inflammatory effect as, for instance, suggested by reduction of circulating *Il6* levels [7-9]. The intranasal infection procedure appears to be a more likely candidate. Despite the relatively small volume of 20 μl that is used and the near physiological properties of PBS, we consider it likely that entry of PBS into the airway creates a stress response similar to that observed after fluid aspiration, including at least focal pulmonary hypoxia due to bronchospasm. Responsible mechanisms may both relate to stimulation of nerve endings in the airway epithelium and direct noxious stimulation of airway epithelial cells. Indeed, except for *Irg1*, three of the four mRNAs whose expression was regulated in response to mock treatment are known to be induced during a stress response or hypoxia at the cellular or tissue level (*Retnla: *[10]; *Il6*: e.g., [11]; *Cxcl10*: [12]). The fourth one, *Irg1*, is preferentially expressed in macrophages, is strongly induced during macrophage activation, and localizes to mitochondria [13,14]. Its expression in stress or hypoxia has not been examined, and it would therefore be interesting to test whether it plays a role in these processes. The four interferon related genes (*Stat1*, *Ifng*, *Ifnl2* and *Mx1)* were clearly induced in infected mice only. They have all been shown to play important roles in the host defense against IAV and other viruses [15-17]. The *Mx1* gene is nonfunctional (truncated) in certain mouse strains including DBA/2J and C57BL/6J, but even the nonfunctional murine form is fully interferon inducible [18], suggesting that it does reflect the anti-influenza interferon response of the DBA/2J and C57BL/6J mice. Among these four genes, only *Stat1* has been shown to be regulated by stress or hypoxia [19,20]. Interestingly, it was not affected by the mock treatment in the presented study, perhaps because its sensitivity to regulation in this murine model is not high enough to respond to any stress/hypoxia due to the mock treatment. Indeed, its upregulation in the infection was much smaller compared to the other three interferon-related genes. Thus, the observation that expression of these four interferon-related mRNAs was not affected by the mock treatment supports the aforementioned notion that the procedure-associated effects in this model relate to a stress response that can be functionally separated from the antiviral response.

### Differences between the two mouse strains

Differences were observed in the magnitude of the response to both mock treatment and viral infection. The fact that both procedure and infection-related responses were more vigorous in the DBA/2J mice agrees with the previously described overall stronger inflammation in this strain during IAV infection [1]. This may reflect a greater intrinsic propensity to inflammation, but also the higher rate of viral replication in this strain. We favor a combination of both models, as the procedure-dependent effects, too, were brisker in the DBA/2J mice.

### Limitations

The relatively small sample size represents a limitation of this study. Nonetheless, statistical significance was reached for several variables. A larger sample size would likely reveal additional significant changes, such as procedure-dependent regulation of *Il1b*, at least in the DBA/2J strain, in which there currently is a tendency toward significance (mean fold increase at 6 h in mock-treated mice = 2.8; p = 0.09). In addition, the small number of target mRNAs does not represent overall gene expression in the lung. Other methods such as RNA deep sequencing would likely reveal genes showing an earlier response to IAV infection or a longer persistence of procedure-dependent effects.

## Conclusions

Despite the aforementioned limitations, the presented results clearly show that the manipulations surrounding the infection procedure can affect pulmonary gene expression in a host strain-dependent manner for approx. 24 h. Thus, “mock treatment” controls should be included in all murine studies on IAV infection where measurements are to be taken within approx. the first 24 h. Likewise, such controls are likely needed in similar studies with other viral and non-viral respiratory pathogens. The degree to which a mock effect might interfere with infection-dependent data would depend on the kinetics of replication and spread of the respective infection in the host animal.

## Abbreviations

FDR: False discovery rate; ffu: Focus forming units; h: Hour; IAV: Influenza A virus; MGI: Mouse Genome Informatics; PBS: Phosphate-buffered saline; qRT-PCR: Real-time reverse transcriptase polymerase chain reaction. Gene name abbreviations are spelled out in the Methods section.

## Competing interests

The authors declare that they have no competing interests.

## Authors' contributions

MP experimental design, animal work, laboratory analyses, graphics, data analysis, preparation of manuscript. MT experimental design, laboratory and data analyses, preparation of manuscript. FK data analysis. KS experimental design, preparation of manuscript. FP experimental design, preparation of the manuscript, supervision of the study. All authors read and approved the final manuscript.
